# Herbalists and Insured Persons

**Published:** 1913-04-19

**Authors:** Gordon P. Ward

**Affiliations:** The National Medical Guild. Pp. General Secretary. Temporary Offices: 34 Villiers Street, Strand, W.C.


*To the Editor of* The Hospital.

### Herbalists and Insured Persons.

*To the Editor of* The Hospital.

Sir,—May I call the attention of your readers to the following facts which show that the State proposes to recognise as medical treatment for which the State will pay and compel employers to pay the ministrations of herbalists, Christian scientists, and other similar persons? The facts are as follows:—

1. On April 1 a representative of the National Medical Guild had occasion to interview an official at the Insurance Commissioners' office. The interview was arranged with regard to other matters, but, in the course of conversation, it transpired that the Commissioners were aware that certain Insurance Committees in the North of England were allowing "own arrangements" under the Insurance Act to be made with herbalists who had no sort of medical training. Allowing these arrangements means, of course, that the Committees in question were prepared to pay for them.

2. On the same day a letter was sent from the National Medical Guild in which, among other matter, appeared the following sentence, viz.:

"May I also state that I am informed that certain Insurance Committees in the North of England are permitting insured persons to make arrangements for medical treatment with herbalists and others not being medical men? May I ask whether this is legal, and if not, by whom and what action should be taken in the matter?"

This letter was sent in order to obtain written verification of the fact.

3. On April 7 a reply was received from the Commissioners in which, again among other matter, appeared the sentence:

"There is nothing in the National Insurance Act or the regulations as to medical benefit which required that an insured person shall only be allowed to make his own arrangements for medical treatment and attendance on condition that these arrangements are made with a duly qualified medical practitioner."

4. On April 10 Sir J. D. Rees, M.P., asked a question in the House on the subject, which was as follows, viz.:

"Whether, under any section or subsection of the National Insurance Act or regulations relating to such Act, the Commissioners will recognise and sanction the medical treatment of insured persons by unqualified individuals, such as herbalists and Christian scientists; and, if so, whether, seeing that herbalists and other unqualified persons cannot administer anæsthetics, perform operations, treat fractures, or give death certificates, he will say why this class of treatment is recognised under the regulations?"

Mr. Robertson replied for Mr. Masterman as follows (this is taken from the official Parliamentary report), viz.:

"There is no provision in the regulations dealing specially with these cases, but, subject to the conditions therein prescribed, it is open to an Insurance Committee to allow insured persons to make their own arrangements instead of receiving medical attendance and treatment from a duly qualified medical practitioner on the panel."

Sir J. D. Rees then asked:

"Can the honourable gentleman answer the question as regards the death certificates? How can Christian scientists give certificates?"

Mr. Robertson replied:

"It will rest with the Insurance Committee, if they are satisfied."

Putting the facts very shortly, they are as follows: We learnt from the Commissioners' office that arrangements with "quacks" were being made. In reply to a letter they said there was nothing to prevent them being made. In the House of Commons they said the same.

We are sending this information to the press because it has a very important bearing on the public health. There are always a certain number of persons who will believe that any man can cure them who says he can. Moreover, in isolated cases, the unqualified practitioner achieves success when the medical man has failed—either he inspires more faith by making extravagant claims, or he uses methods which fail miserably in 99 per cent. of cases (which one only hears about when they come to medical men for treatment subsequently, they do not get into the press), and which medical men know to be unjustified in any but the most occasional cases. The State has never before definitely assisted the delusions of the unfortunate dupes of unqualified practice; now it does so. Are the public satisfied that the State should require five years' training before a medical man may practice medicine, but, at the same time, pay out of public funds for the dangerous ministrations of the unqualified, and leave it, in the year of grace 1913, to Insurance Committees to say whether a Christian scientist's certificate is legal? If they are not, we appeal to them to see that their representatives on Insurance Committees do not countenance this sort of thing or to inform us of any that do in order that we may take definite action in the matter.—We are, Sir, yours faithfully,

The National Medical Guild.
Gordon P. Ward,
Pp. General Secretary.

Temporary Offices:
34 Villiers Street, Strand, W.C.
April 14, 1913.

[This letter should be compared with an official statement contained in our Notes this week on the subject of herbalists.—Ed. The Hospital.]

